# More Than Just a Barrier: The Immune Functions of the Airway Epithelium in Asthma Pathogenesis

**DOI:** 10.3389/fimmu.2020.00761

**Published:** 2020-04-28

**Authors:** Andreas Frey, Lars P. Lunding, Johanna C. Ehlers, Markus Weckmann, Ulrich M. Zissler, Michael Wegmann

**Affiliations:** ^1^Division of Mucosal Immunology and Diagnostics, Research Center Borstel, Borstel, Germany; ^2^Airway Research Center North, German Center for Lung Research (DZL), Borstel, Germany; ^3^Division of Asthma Exacerbation & Regulation, Research Center Borstel, Borstel, Germany; ^4^Division of Experimental Pneumology, Research Center Borstel, Borstel, Germany; ^5^Department of Pediatric Pulmonology and Allergology, University Children’s Hospital, Lübeck, Germany; ^6^Center of Allergy & Environment (ZAUM), Technical University of Munich and Helmholtz Center Munich, German Research Center for Environmental Health, Munich, Germany; ^7^Member of the German Center for Lung Research (DZL), CPC-M, Munich, Germany

**Keywords:** asthma, inflammation, barrier, mucus, polarization, imprinting, trained immunity

## Abstract

Allergic bronchial asthma is a chronic disease of the airways that is characterized by symptoms like respiratory distress, chest tightness, wheezing, productive cough, and acute episodes of broncho-obstruction. This symptom-complex arises on the basis of chronic allergic inflammation of the airway wall. Consequently, the airway epithelium is central to the pathogenesis of this disease, because its multiple abilities directly have an impact on the inflammatory response and thus the formation of the disease. In turn, its structure and functions are markedly impaired by the inflammation. Hence, the airway epithelium represents a sealed, self-cleaning barrier, that prohibits penetration of inhaled allergens, pathogens, and other noxious agents into the body. This barrier is covered with mucus that further contains antimicrobial peptides and antibodies that are either produced or specifically transported by the airway epithelium in order to trap these particles and to remove them from the body by a process called mucociliary clearance. Once this first line of defense of the lung is overcome, airway epithelial cells are the first cells to get in contact with pathogens, to be damaged or infected. Therefore, these cells release a plethora of chemokines and cytokines that not only induce an acute inflammatory reaction but also have an impact on the alignment of the following immune reaction. In case of asthma, all these functions are impaired by the already existing allergic immune response that *per se* weakens the barrier integrity and self-cleaning abilities of the airway epithelium making it more vulnerable to penetration of allergens as well as of infection by bacteria and viruses. Recent studies indicate that the history of allergy- and pathogen-derived insults can leave some kind of memory in these cells that can be described as imprinting or trained immunity. Thus, the airway epithelium is in the center of processes that lead to formation, progression and acute exacerbation of asthma.

## Introduction

With more than 300 million people affected bronchial asthma is one of the most common chronic inflammatory diseases worldwide (1). Actually, 1 out of 250 deaths is associated with asthma and it causes annual direct medical (drugs, care, hospitalization) and indirect economic (productivity loss, early retirement) costs of about €34 Billion for the EU (2) and over 80 Billion for the United States (3, 4), making it a major burden for public healthcare systems (5).

Asthma is characterized by acute broncho-obstruction, in combination with additional symptoms such as cough, chest tightness, shortness of breath, and wheezing, which vary in extent and over time. These symptoms arise on the basis of chronic airway inflammation in response to a trigger, most commonly inhaled allergen(s), that causes airway hyperresponsiveness (AHR), airway remodeling and mucus hypersecretion (6). Due to the complexity and variation of the symptoms along with its pathogenesis, asthma is nowadays described as a heterogeneous syndrome with distinct sub- or endotypes. However, the majority of asthma patients displays allergic inflammation of the airways, which can be classified by profiles of several characteristic mediators in “TH2 high” or “TH2 low” subtypes (7). Thus, in sensitized individuals T helper 2 (TH2) cells orchestrate allergic inflammation by releasing a typical array of cytokines including interleukins (IL)-4, -5, -9, and -13 and granulocyte-macrophage colony stimulating factor (GM-CSF). These mediators induce the production of allergen-specific immunoglobulin (Ig) E, TH2 cell development, goblet cell differentiation, submucosal gland activity, as well as recruitment, maturation, and activation of eosinophils and its precursors (8). Activation of mast cells and eosinophils via IgE-bound allergens results in their degranulation and, thus, in the release of a plethora of effector molecules and growth factors that on the one hand destroy airway tissue and on the other hand conduct its repair. Chronic activation of these processes ultimately lead to signs of airway remodeling such as increased smooth muscle mass, subepithelial fibrosis, and epithelial desquamation, which in turn give rise to the pathological changes and clinical symptoms characteristic for asthma (9).

Allergic sensitization against aeroallergens represents the strongest factor predisposing for the development of asthma, indicating a hyperreaction of the immune system to be the central event within the pathogenesis of this disease. Nevertheless, structural cells and particularly airway epithelial cells also appear to be of critical importance. This is not surprising since these cells represent the barrier that first encounters environmental stress factors like air pollutants, bacterial and viral pathogens, as well as allergens, and markedly contributes to their neutralization by a mechanism called mucociliary clearance (MCC). Besides these barrier and cleaning functions airway epithelial cells also exert a number of immunological tasks interweaving the role of the epithelium with that of the above-mentioned cells of the immune system. Here we aim to review these immune functions of the airway epithelium against the background of asthma pathogenesis.

## The Barrier Function of the Airway Epithelial Cell Layer Itself

The main purpose of mucosae is to separate the body from its environment and therefore they are essential for the maintenance of the inner homeostasis. Though this task is not commonly regarded as an “active” or “typical” immune function, it is absolutely central for the defense against allergens, pathogens and other harmful environmental factors. In order to fulfill this function the airway epithelium forms a continuous, self-cleaning barrier with a considerable resistance against biological, chemical or physical stressors (10). Together with the physical barriers of the MCC and glycocalyx, this is achieved by three types of intercellular epithelial junctions that form the structural adhesion forces of the airway mucosa by linking the intracellular structures of the cytoskeleton of one epithelial cell to that of its neighbors. These junctions involve adherens junctions (AJs), hemidesmosomes, and tight junctions (TJs).

AJs can appear as spots (adhesion plaques) or as bands encircling the cell (zonula adherens). In the junctional zone AJs interconnect the actin filaments of the adherent cells via homotypic transmembrane E-cadherin adhesions and anchor proteins like actinin, vinculin, and α-, β-, and p120 catenins, while adhesion plaques attach the cells to the extracellular matrix (11).

Similarly, hemidesmosomes are focal structures that form adhesive bonds between the cytoskeleton of epithelial cells and the lamina lucida, which is a part of the lamina propria. Hemidesmosomes utilize integrin α6β4, plectin 1a and the tetraspanin CD151 connecting laminin and fibronectin of the extracellular matrix to the intermediate filaments of the cytoskeleton (12).

In contrast, TJs form a multiprotein junctional complex called zonula occludens (ZO) that in turn appears as the main regulator of the paracellular permeability. These complexes are formed by several transmembrane and cytoplasmic proteins that are attached to actin filaments of the cytoskeleton. The main components of TJs are claudins and occludins, proteins with four transmembrane domains, as well as so-called junctional adhesion molecules (JAMs) belonging to the immunoglobulin superfamily with only one transmembrane domain. These proteins are connected to actin filaments by cingulin and ZO proteins 1, -2, and -3 (13).

In the airways of healthy individuals, the TJs of the zonula occludens and AJs of the zonula adherens constitute dense protein networks that interconnect the basolateral sides of epithelial cells in such a way that they prevent the paracellular passage of basically all molecules, including water, ions and proteins, as well as of pathogens or other inhaled particulate matter. Several findings strongly indicate that in asthma patients the barrier function is impaired by epithelial disruption. For example, endobronchial biopsies revealed a fragile or even injured airway mucosa with partially or completely uncovered areas and detachment of columnar, ciliated cells (14). Epithelial desquamation is further indicated by the presence of epithelial cells in bronchoalveolar lavage (BAL) and of creola bodies (epithelial cell aggregates) in sputum of asthmatics (15). Furthermore, bronchial biopsies of asthmatic subjects displayed patchy disruption of TJs (16) and the expression of a number of proteins that are essential for the formation of TJs and AJs has been shown to be markedly reduced. Among these proteins are α-catenin (17), β-catenin (18), occluding (16), ZO-1 (16, 17), and E-cadherin (17, 19). The levels of the latter one in sputum also correlate with asthma severity (20). These data are further supported by *in vitro* studies where primary bronchial epithelial cells are kept in air liquid interface (ALI) culture, a method that allows the cells to differentiate and form a pseudo-stratified epithelial monolayer largely resembling the physiological structure of the airway mucosa. Once this structure has been established, *in vitro* barrier integrity can be assessed by measuring the transepithelial electrical resistance (TEER), a characteristic that is indicative of the tightness of a cell layer (21). Several studies showed that ALI cultured airway epithelia from asthma patients display a decreased TEER in comparison to epithelia derived from healthy controls (16, 22, 23).

## Impairment of Cellular Barrier Functions in Asthma Pathogenesis

To date, three different factors are discussed to have a harmful impact on the barrier integrity of the airway epithelium in asthma pathogenesis: allergens themselves, viral infection, and (allergic) inflammation. According to the “protease hypothesis” allergens with an inherent protease activity are capable of cleaving the protein components of the aforementioned intercellular epithelial junctions so that the barrier function is disrupted and allergens can penetrate the airway mucosa on the paracellular route, which eventually could result in sensitization against them. Accordingly, a considerable number of allergens has been tested *in vitro* for proteolytic potential and for an effect on epithelial barrier integrity. Several studies provided evidence for a direct cleavage of e.g., occludin and ZO-1 proteins by the major allergen from house dust mites (*Dermatophagoides*), Der p 1 (24, 25). House dust mite extracts as well as Der p 1 have been shown to increase the permeability and to decrease TEER of epithelial layers *in vitro* (23, 25, 26). Comparable effects have been shown for extracts of the allergenic fungus *Alternaria alternata* that reduced TEER of human bronchial epithelial cells *in vitro* (27) or the *Aspergillus fumigatus-*derived alkaline protease 1 (Alp-1) (28). Similarly, a variety of different pollen extracts has been investigated for their effect on the barrier integrity of epithelial cells *in vitro*. Diffusates of Italian cypress (*Cupressus sempervirens*), Orchard grass (*Dactylis glomerata*), Olive (*Olivia europaea*), and *Scots pine* (*Pinus sylvestris*) have been shown to affect claudin-1, E-cadherin, and occludin expression and thus to disrupt epithelial junctions in ALI cultures of Calu-3 cells, an effect which could be suppressed by protease inhibitors (29). Japanese hop (*Humulus japonicus*) extract also reduced expression of occludin in a comparable setting (30). Another study provided evidence for proteolytic activity of Giant ragweed (*Ambrosia trifida*), Kentucky bluegrass (*Poa pratensis*), and White birch (*Betula pendula*) as shown by reduced expression of claudin-1, occludin, and ZO-1 in Calu-3 as well as in MDCK cells (31).

However, an inherent protease activity appears not to be the only way, by which allergens can impair the barrier integrity of the airway epithelium. Cockroach, HDM, fungus, and mold extracts have also been shown to activate the protease-activated receptor (PAR-) 1 and/or 2, which in turn leads to degradation of AJ components (25, 32–34).

The effect of viral infections on airway barrier function is even more pronounced than that of allergens. Respiratory viruses cause junction dysfunction by different mechanisms: human rhinoviruses (HRV), respiratory syncytial virus (RSV), human metapneumovirus (HMPV), influenza and parainfluenza viruses bind to their entry receptor, which are typically protein or sugar structures expressed on the cellular surface for other purposes, leading to endocytosis of the virus. Once the virus has been internalized, it uncoats and initiates the viral replication process, which has certain consequences for infected cells. On the one hand, the cell starts with the production of type I interferons (IFN) in order to slow down the internal virus replication and to activate the cellular immune response against the virus. In consequence, infected airway epithelial cells are killed by virus-specific, cytotoxic CD8 + T cells. On the other hand, the virus itself also kills epithelial cells, since it induces morphological alteration of the cells summarized as cytopathic effect (CPE). For HRV and influenza viruses, the CPE manifests in rounding and detachment of airway epithelial cells that are ultimately lysed by the virus in order to release freshly produced viruses. Paramyxoviruses such as RSV and HMPV induce cell fusion so that four or more cells form typical syncytia (35, 36). Additionally, at least HRV and RSV affect the barrier integrity of the airway epithelium by reducing the expression of epithelial junction proteins (37–40). It could be shown that HRV increases epithelial permeability by a reduction of occludin and ZO-1 expression (41, 42). RSV also disrupts junctional complex structures by fostering the activity of protein kinase D (PKD) (43).

The antiviral immune response also includes the release of cytokines that directly affect epithelial barrier function as well. This is especially true for IL-1β, IFN-γ and tumor necrosis factor (TNF). These cytokines have been shown to support epithelial permeability and to decrease expression of claudins, JAM, occludin, and ZO-1 in several *in vitro* studies (44–46). In case of asthma, these effects are even more pronounced because of the allergic inflammatory response that already exists before the viral infection of the airway epithelium. Hence, TH2 type cytokines like IL-4 and IL-13 also increase barrier permeability by inhibiting the surface expression of β-catenin, E-cadherin, occludin, and ZO-1 (45, 47). In addition to cytokines, mast cell derived mediators also appear to have an effect on the barrier function of the airway mucosa. Histamine for example has been shown to contribute to transient disruption of apical junctional complex integrity and thus to increase epithelial permeability *in vitro* (48).

Allergens, viruses, and the inflammatory response to their exposure represent extrinsic factors that impair the barrier integrity of the airway epithelium. However, some studies suggest that epithelial cells of asthma patients inherently predispose for an increased permeability. As already mentioned above, airway epithelial cells that have been isolated from asthmatics and propagated *in vitro* to form an epithelial monolayer under ALI culture conditions, display a decreased TEER as compared to cells from healthy donors (23, 45). This observation indicates that the cellular properties leading to an increased barrier permeability are somehow imprinted within the cells. Whether this is a matter of genetic predisposition encoded in epithelial stem cells or whether epithelial cells from asthma patients “remember” previous insults by epigenetic modifications that predispose for asthma development in later life remains elusive.

## Passive Luminal Barrier Structure on the Airway Epithelium

Besides the barrier function of the epithelium provided by the mere presence of the sealed cell layer itself, two additional barrier structures are “exported” onto the luminal surface by the airway epithelium, a static one dubbed glycocalyx or periciliary layer (PCL) and a mobile one termed mucus.

## Structure and Function of the Periciliary Layer

**FIGURE 1:**
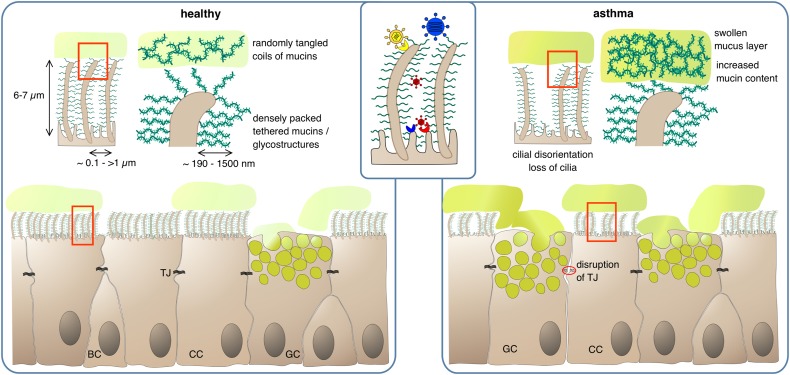
Protection of epithelial surfaces by physical barriers. In the healthy state, ciliated cells (CC) form a tight epithelial layer where paracellular passage is prevented by sealing of lateral intercellular spaces with tight junctions (TJ). The apical epithelial cell surface, including the cilia, is covered by a layer of membrane-anchored glycoproteins and glycolipids, the glycocalyx. The dense meshwork of glycostructures restricts access of luminal matter to the apical cell surface; depending on their size, larger pathogens can be cut off from their receptor if it is not present on cilia (inset). Goblet cells (GC) secrete mucus, consisting of highly glycosylated mucins which absorb large quantities of water to form a viscous gel. The mucus – and any matter trapped within – is transported upward in the airway lumen by the coordinated beating of the cilia. BC, basal cells. In the asthmatic state, barrier functions can be compromised by partial disruption of tight junctions and gaps in the PCL/glycocalyx meshwork due to loss of cilia. Mucus clearance is impeded by increased mucus viscosity and swelling of the gel matrix, and by disturbance of ciliar beating due to disorganization and dykinesia of cilia.

The glycocalyx or PCL is a sponge/fleece-like, cell membrane-anchored layer of glycolipids and glycoproteins – mainly mucins (see below) – that vertically stick out of the apical epithelial cell membrane. Although mainly attributed to the gut epithelium where it can extend up to 1500 nm (49) and to the vascular endothelium (50, 51) a glycocalyx/PCL is also present throughout the airway epithelium even down to the alveoli (52), again with heights up to 1500 nm in certain areas (53) (Figure 1). This static glycoprotein and glycolipid coat stores water to control mucus hydration but also serves as a protective zone against the compression of the mucus lying above in order to allow persistent cilia beating for ongoing functionality of the mucociliary clearance (MCC; see below) (53, 54). Beyond that, the glycocalyx/PCL regulates receptor specificity by architectural means and prevents the progression of viruses through the occasionally patchy mucus layer. It has been shown that the height and density of the epithelial glycocalyx can determine whether a ligand-equipped nanoparticle may attach to its membrane receptor or not (49, 55). In line with this, the inefficiency of adenovirus-mediated gene transfer into the airway epithelium was found to be caused by the membrane-tethered glycocalyx/PCL proteins that put a halt to the advance of the viral vectors (56, 57). Consequently, the susceptibility of the airway epithelium toward infection is at least to some extent controlled by the glycocalyx/PCL. Very small viruses such as bocavirus (HBoV1) and HRV, which are 20–30 nm in size (58, 59) should readily advance through the PCL to the epithelial plasma membrane as has been observed with nanoparticles of the respective size (53). Consequently, those viruses should be able to luminally infect airway epithelial cells as long as their receptor is present on the apical side. Little is known about receptor distribution *in vivo* but at least on cultured airway epithelial cells apical receptor expression and/or infectivity has been demonstrated for both viruses (60–63). Larger particles of about 100 nm and above, on the other hand, are efficiently blocked by the PCL (53). Hence, viruses such as RSV, HMPV, influenza and parainfluenza viruses, adenovirus or coronavirus, which are in this size range (64–67), should be hindered efficiently by the PCL to infect the host. Yet, those viruses often are associated with respiratory infections and asthma exacerbations (68, 69). One possibility for them to infect the airway epithelium may be the presence of the viral receptor on structures that extend from the PCL such as the tips of the cilia. An example for this is chemokine receptor CX3CR1 via which RSV can infect its host. In differentiated human airway epithelial cells this molecule is highly abundant on cilia (70, 71). Another possibility is the preceding action of a door-opener such as HBoV1 which may pave the way for further viral infections. HBoV1 was shown to persist for several months in the human airway epithelium (72) and causes pyroptotic cell death, epithelial cell hypertrophy, loss of cilia and disruption of the tight junction barrier (60, 61). Such a predamaged epithelial barrier may then readily fall victim to an influenza, parainfluenza or HPMV infection. With up to 13% of asthma exacerbations in small children found to be associated with HBoV1 infection (73) it may be worthwhile to further investigate possible coinfections with HBoV1 in asthma exacerbation cases. In this context, it may also be of interest that the treatment of chronic inflammatory diseases of the airways such as asthma with corticosteroids (CS) seems to reduce the glycocalyx/PCL height on the alveolar epithelium (74) thereby rendering the lung more susceptible to e.g., *Pneumocystis carinii* infection. Consequently, alleviating chronic inflammation in asthma with CS may make the patient more susceptible to certain infections, which in turn may enhance inflammation again, clearly a two-edged outcome of CS therapy in asthma.

Although the glycocalyx appears to be static on the architectural level, it may not be invariant in terms of its molecular composition. It was shown that lipopolysaccharide exposure could lead to heparan sulfate shedding from the airway epithelium thereby causing increased lung permeability (52). Allergen exposure of experimental animals resulted in different glycosylation patterns of the glycocalyx (75), which may result in a deviant presentation of viral and bacterial receptors on the cell surface. In light of the above, the airway epithelial glycocalyx seems to play a so far underestimated but possibly important role in airway epithelial defense. Whether or not the molecular composition of this static cell coat is different in asthma, remains to be investigated.

## Composition and Function of Mucus

While the role of the glycocalyx/PCL is still subject of debate, the importance of mucus in airway luminal defense is unchallenged. Mucus is an unstirred discontinuous sheet of secreted mucous hydrogel which floats on top of the epithelium and is transported toward the oral cavity like the cargo on a conveyor due to the constant and coordinated beating of underlying ciliated cells (76). The mucus carried upward by this mucociliary clearance (MCC) mechanism can be swallowed or expectorated. As the mucus layer separates the airway lumen from the epithelium only objects that diffuse faster “vertically” toward the epithelial surface than the mucus is transported “horizontally” toward the oral cavity can reach the epithelial cell membranes. Thus, only nanoscalar objects and smaller, like gases, water, salts and nutrients are able to reach the epithelial cells (77, 78). This way the mucus carpet provides a protective line of defense against pathogens, dust and other harmful objects that might be inhaled by an individual. In addition to that, the sticky texture of the mucus slows down airborne objects and further prevents their advance to the epithelial cell layer. In order to exert these functions properly, mucus requires a specific composition. Mucus consists mainly of water, further components are salts, lipids and proteins. Among those, antimicrobial proteins like lysozyme, immunoglobulins and antimicrobial peptides are major molecular scavengers distributed within the mucus layer (79–81). The characteristic viscous, elastic and sticky properties of the mucus are provided by a group of macromolecules named mucins (82, 83).

To date, 21 genes coding for mucins have been described (84). Their protein products are secreted either to form mucus or remain immobile on the apical membranes of the airway epithelial cells where they become part of the glycocalyx/PCL. MUC5AC and MUC5B are the major secreted mucins in the airways. In addition, MUC2 and MUC19 are also part of airway mucus, albeit to a considerably smaller proportion, and thus belong to the family of secreted mucins (83). In contrast, MUC1, MUC4 and MUC16 are tethered to the cells of the airway epithelium (Figure 1) thereby contributing to the static luminal epithelial barrier, which resides underneath the mobile mucus layer (85, 86).

The viscous and elastic properties of the mucous gel are primarily given by the secreted polymeric mucins MUC5AC and MUC5B (83). These mucins are highly O-glycosylated proteins enriched with amino acids like proline, serine or threonine (87). Although both have a similar structure, MUC5B and MUC5AC differ in charge due to differential glycosylations (88). Their production depends on cell type and site of production. In the upper airways, MUC5AC is produced by epithelial goblet cells while MUC5B is secreted from mucous cells in submucosal glands from secretory cells in the tracheal and bronchial epithelium. In the distal airways, MUC5B is also produced by secretory cells of the epithelium and seems to be the major mucin of this airway region (83, 89, 90). Before secretion, polymeric proteins are stored in secretory granules in a compacted, dehydrated state. Upon release, they switch to a hydrated form, which is necessary for the mucous gel-like properties (91, 92). Whether the two different mucins have different functions restricted to their site of production or whether the two mucins mingle to create a novel type of barrier structure is not clear yet. At least some studies analyzing airways of piglets have shown that MUC5B strands are becoming coated with MUC5AC to some extent after release at the epithelial surface (93, 94). A possible role of MUC2 and MUC19 has not been identified yet.

Howsoever, under healthy conditions, the viscous mucus traps noxious substances, which are then removed from the airway via cilial beating by the MCC (95). In asthma, the MCC is impaired leading to mucus plug formation which in turn results in the characteristic obstruction observed in asthmatics. This is already a feature of mild stable asthma and the dysfunction worsens during aggravation of asthma and in asthma exacerbations (96–98). One reason is an increased mucin content of the mucus thereby disturbing its regular composition. Normally, the airways’ mucus consists of ∼98% water and only ∼2% solid factors mainly mucins. In obstructive diseases, the amounts of mucins rise up to 8–15% (54, 86). Due to its hygroscopic nature, this leads to acquisition of water from the underlying PCL/glycocalyx and shrinking of this static layer. The now protruding cilia either project into the mucus or get bend (54). Both effects impede passing on of the mucus to the next cell. Loss and/or disorientation of cilia as it is typical for asthmatics will further disturb the “bucket chain”-like transport process (99). On the cargo side enhanced intermolecular crosslinking of mucus constituents by oxidative processes may further complicate forwarding. It will also increase mucus viscosity eventually leading to plug formation. Oxidative intramolecular crosslinking of biomolecules is predominantly caused by cysteines whose thiol side chains can form disulfide bridges. All mucins are rich in cysteines, especially in their less glycosylated carboxy- and amino terminal regions. In the “normal” mucous gel of healthy individuals these cysteines are believed to be only moderately crosslinked, forming a lightly entangled network. In asthma, however, the degree of crosslinking and the density of the mucin network increases considerably (100, 101) (Figure 1). Increased oxidative stress appears to play an important role in this respect with eosinophils being the main suspects for oxidant production. The abnormally high levels of eosinophil peroxidase detected in the sputum of asthma patients may form an oxidative milieu. This would also bring the widely observed correlation between airway eosinophilia and airway obstruction into a causative relationship (102). Lastly, the MUC5AC of asthmatics tends to tether to the epithelium, which also complicates mucus forwarding (103).

In asthmatics not only the amount but also the composition of the mucus changes, especially the ratio of MUC5AC to MUC5B as well as the posttranslational modification of MUC5B. Mucus from healthy individuals contains predominantly MUC5B, which is essential for the MCC and protection against pathogens (104–106). In asthma, the proportion of MUC5B relative to MUC5AC often decreased (104, 105) along with the expression of a low-charge form of MUC5B. Consequently, there was a changed ratio between the two differently glycosylated forms of MUC5B (104, 107). The importance of MUC5B is indicated by *Muc5b*-deficient mice, which showed an accumulation of undesired substances e.g., bacteria, resulting in severe inflammation and airway obstruction (106).

The ratio between MUC5B and MUC5AC changes dramatically in asthma because MUC5AC expression and protein production are substantially upregulated in asthmatic patients (104, 105, 107). Especially patients with an eosinophilic type 2 asthmatic phenotype showed a shifted ratio toward higher MUC5AC concentrations (105, 108). This is in line with the assumption that MUC5AC seems to be important for the defense against enteric nematodal and influenza infections (109, 110). The increased expression of MUC5AC seems to depend on substantially increased levels of IL-13. The IL-13 signaling pathway activates the signal transducer and activator of transcription 6 (STAT6), which induces the expression of MUC5AC via various regulators (111) and appears to be involved in AHR development (112, 113). Several studies using *in vitro* systems with human epithelial cells or murine models validated this mechanism (114–117). Furthermore, EGFR, which is also overexpressed in asthma, also induces the expression of MUC5AC (118–121). This excessive production of mucins in the asthmatic airway epithelium leads to an increased volume of intracellular stored mucins, a mucus metaplasia (122). Thus, a higher number of goblet cells compared to the healthy situation appears in case of asthma (122). It is not exactly understood, whether this switch from a muco-ciliary phenotype to a mucous metaplastic phenotype develops from goblet cell hyperplasia, metaplasia or both as reviewed before (123). In animal models, goblet cell metaplasia/hyperplasia arises from an increased expression of primarily IL-13, but also of IL-4 and IL-9 (124–127). These cytokines are highly upregulated in asthmatic individuals (128–131). One important factor for the development of the goblet cell metaplasia is Notch2 regulated by IL-13 (132). Studies analyzing the function of SAM-pointed domain containing ETS transcription factor (SPDEF) highlighted its essential role in the development of goblet cell differentiation, hyperplasia and mucous metaplasia (133–135). Therefore, SPDEF seems to inhibit the expression of Forkhead box protein A2 (FOXA2) which is an important negative regulator of genes associated with mucous metaplasia and goblet cell hyperplasia (111, 121, 133, 136, 137).

Thus, the physical barriers provided by the airway epithelial layer seem to be deeply disturbed in asthmatic individuals. Although mucin is one of the most important barrier molecules in the airways its unbalanced overproduction is clearly detrimental to the desired outcome. Mucus plugging impedes egress of the active luminal defense molecules necessary to eliminate invaders.

## Active Luminal Defense Mechanisms on the Airway Epithelium

Although strong walls (tight junctionally sealed epithelial cell layer) surrounded by a glacis (pericilial layer/glycocalyx) and a moat filled with flowing liquid (MCC) are crucial to prevent invaders from entering a castle, active defenses are necessary to end the siege. This is of particular importance when the besieger can replicate and thus may increase continuously by number, as is the case when pathogenic bacteria colonize the luminal side of the airway epithelium.

## Defensive Molecules Produced by the Epithelium

In order to get rid of a potential invader the airway epithelium possesses a battery of defense molecules, with which a potential microbial enemy can be attacked, destroyed or removed out of the airway lumen. Prominent innate molecular scavengers are lysozyme, transferrin and antimicrobial peptides. Lysozyme is produced in large amounts (20 mg/day) by serous cells of the upper human airway epithelium (138) and is able to destroy the polysaccharide capsules of many bacterial species. It has been shown that the production of lysozyme by serous cells residing in the serous glands of the upper airways is crucial for defending against bacterial airway invaders (139). Once the polysaccharide capsule is destroyed or damaged, so called defensins or antimicrobial peptides may exert the lethal hit to the invader. Defensins can form holes or pores into a bacterial cell membrane thereby killing a pathogen that aims to enter the body (79, 140, 141). In addition, lactoferrin is produced and secreted by serous cells (142), and transferrin is expressed by alveolar type I cells (143). These ferrins are iron-binding proteins, which deplete their environment from iron ions that are essential for the growth of a self-replicating organism (143, 144). Consequently, the pathogen is starved out.

## The Role of Secretory IgA in Epithelial Defense

**FIGURE 2:**
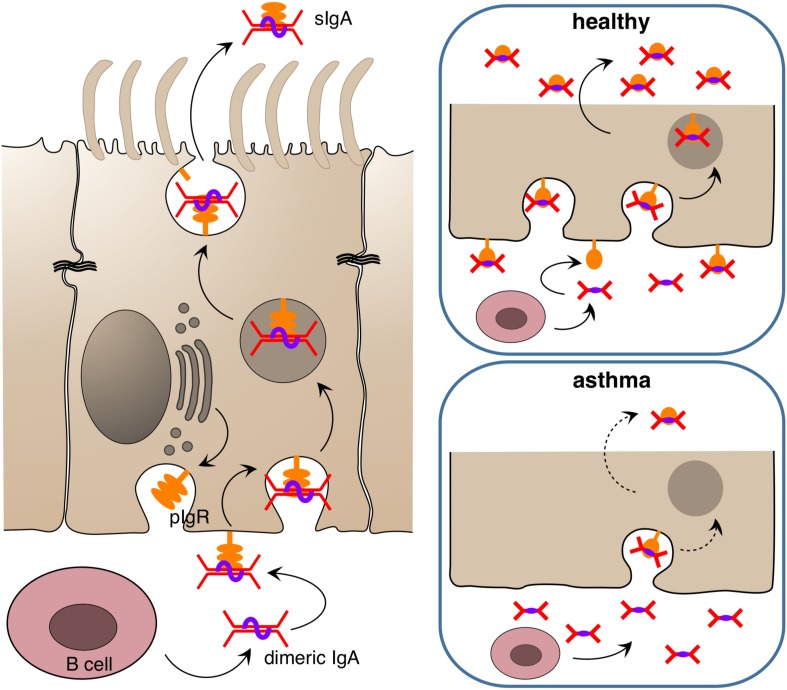
Release of immunoglobulin A at epithelial surfaces. Dimeric IgA is released submucosally by B cells and binds to the poly Ig receptor (pIgR) which is found in high amounts in the basolateral membrane of the epithelium in the healthy state. The complex of pIgR and dimeric IgA is transported in secretory vesicles to the apical side, where it is released into the lumen as secreted IgA (sIgA) to bind target antigens/allergens/pathogens and entrap them in mucus. In the asthmatic state, the amount of pIgR in the epithelium appears to be reduced, resulting in diminished secretion of sIgA and futile accumulation of IgA in the subepithelial compartment.

Besides this innate “rapid response team,” the polarized epithelium of the human airways is also able to transport and apically release immunoglobulins that carry a J-chain (joining chain) by using its poly Ig receptor (pIgR) (145–147) that is expressed by all non-stratified epithelial cells (Figure 2). Only IgM and multimeric IgA are equipped with J-chains (148, 149). These two immunoglobulin classes not only circulate in the bloodstream but are also produced directly underneath the airway epithelium by B cells, given those lymphocytes express the J-chain (150, 151). Functionally, IgM can substitute for multimeric IgA. For that reason IgA-deficient individuals do not show a strong phenotype concerning susceptibility to infection. Nevertheless, secreted IgA (sIgA) outperforms IgM in terms of mucosal protection as it usually displays a higher affinity toward its antigen and, more importantly, is able to crosslink with mucins upon target binding (152, 153). This way an incoming viral or bacterial pathogen becomes trapped in mucus and is removed from the airway surface via the MCC. The protective function of secreted IgA has been demonstrated with various model systems, both for the gastrointestinal mucosa as well as for the airways, using passively administered monoclonal IgA (154–157), injected hybridoma cells whose target specific, dimeric IgAs are then transported across the mucosae (“backpack tumor model”) (158–160) and by neutralization of preexisting mucosal IgA immunity with mucosally administered anti-IgA immunoglobulins (80). Although adaptive multivalent target binding via its hypervariable regions is probably the main mode of protection in those models, sIgA is also able to bind in an innate manner to luminal pathogens via its carbohydrate components by presenting decoy structures that mimic target cell surface receptors (161). If both modes of repelling fail and a pathogen has nonetheless invaded an epithelial cell, dimeric IgA may still be able to protect the infected cell, this time from inside. This is possible whenever the respective pathogen does not directly infect the cytosol of its target cell or inject its nucleic acids directly into the cytosol but rather uses an initial endocytosis step for infection. Depending on the infected organelle, vesicles, which concurrently translocate IgA toward the apical site, may fuse with the infected organelle, bind to the invader and carry it away into the lumen. In addition to this removal activity, mucus crosslinking and the tricking of pathogens by offering decoy receptors, sIgA also scavenges IL-8 and thereby inhibits IL-8-driven neutrophil chemotaxis (162).

In addition to these molecular interactions with a pathogenic target, IgA also binds to numerous cell types that patrol at the airway epithelium. The most important cellular partner seems to be the eosinophil as this cell possesses a total of five different receptors for IgA: FcαRI (CD89), transferrin receptor (TfR) (CD71), pIgR, asialoglycoprotein receptor (ASGPR) and a receptor for secretory component (SCR) with the integrin Mac-1 (CD11b/CD18) serving as a coreceptor for FcαRI (163, 164). Depending on the receptor addressed and the form of IgA offered, i.e., soluble versus target-bound and cross-linked, eosinophils are either calmed down or activated (165–167). Yet, eosinophils are not only manipulated by IgA, they also influence IgA production themselves (168, 169). Thus, immunoglobulin A and eosinophils share a really intimate relationship.

Equipped with less receptors but still responsive to IgA are neutrophils, dendritic cells, macrophages, basophils and even epithelial lining cells that express the transferrin receptor such as alveolar-type 2 cells (170). An additional, so far unidentified receptor is present on M cells (microfold cells) (171, 172). M cells are a specialized epithelial lining cell type that is responsible for antigen sampling at mucosal surfaces and predominantly occurs in the epithelium above organized mucosa-associated lymphoid tissue (173, 174). This receptor senses the distance between two heavy chain domains in IgA. Thus, it is not able to bind IgA1, an IgA subclass present only in primates. IgA1 is different from IgA2 in that it contains a mucin-like, highly glycosylated extension of its hinge region. It is believed that this hinge region also serves as a ligand for yet other IgA receptors (163). If this holds true, subclass switching may be another adjusting wheel for IgA function. The class switch from IgA1 to IgA2 depends on the presence of the cytokines APRIL and BAFF which were shown to be produced by the epithelial layer itself, at least in case of the gut, upon bacterial stimulation (175). This way the microbiome as sparring partner of the epithelium comes into play as the true master of this adjusting wheel. In contrast to the gut where IgA2 prevails, IgA1 is the predominant IgA subclass in the airways (176). This, however, does not imply that IgA2 is of less importance for airway defense. IgA1 simply may be the first class formed after pathogen challenge. Those initial secretory IgA (sIgA) responses are believed to be not very mature. Upon pathogen challenge the human body apparently rapidly switches its current IgM repertoire to IgA even if most of such “first line of defense” IgA are of low affinity (177). This can be regarded as just a “better than nothing” attempt; yet it creates a window of opportunity for the body to develop more powerful sIgA via affinity maturation. Such optimized immunological scavengers are then able to block and eventually clear a microbial infection. Thus, the IgA system in which the transporting epithelium plays a key role is a complex defense machinery that combines innate with adaptive immune responses. It is therefore not surprising that the role of secretory IgA attracted attention in asthma research in recent years.

## sIgA in Chronic Inflammation of the Lung

The role of sIgA in chronic inflammatory lung diseases is still ambiguous. Some studies show that sIgA is necessary to maintain immune homeostasis, other reports claim that sIgA may play a detrimental role in asthma. A harmful effect of IgA in asthma may be explained by its ability to activate eosinophils and neutrophils because both cell types play a central role in the pathogenesis and persistence of asthma (178). When IgA is able to activate those cell types, this would readily lead to the hypothesis that in case of allergic asthma, allergen-specific IgA is responsible for this activation upon allergen exposure. This assumption is supported by the finding that increased levels of both, allergen-specific IgE and IgA were observed in the airway mucosa of patients with atopic asthma and/or rhinitis (179–183), and it was shown that allergen-specific IgA levels were positively correlated to eosinophil activation marker release after segmental lung challenge of asthmatic patients (166). Yet, coincidence and correlation do not necessarily imply a causative relationship. In the abovementioned study, where a positive correlation of allergen-specific IgA and eosinophil activation was observed, the non-allergic control patients also displayed allergen-specific IgA in their airways; but in contrast, they did not have any allergen-specific IgE as was the case for asthmatics. Either so the allergen-specific IgE was responsible for eosinophil activation in asthmatics or the eosinophils of asthmatics underwent some kind of imprinting or immune training that rendered them more sensitive to allergen-specific IgA. With the expression of five different IgA receptors on the eosinophil described so far (163), locked-in differences in IgA receptor expression in eosinophils of asthmatics versus healthy individuals are not impossible. On the other hand, a coincidence of allergen-specific IgA and IgE does not necessarily imply a pathological role of IgA either. It may still be the case that IgA are beneficial to chronic airway inflammation, and the concomitant production of allergen-specific IgA can also be interpreted as a rescue attempt of the body to counteract the allergen-specific IgE.

In fact, more evidence points toward a beneficial role of IgA in asthma and other chronic airway inflammations. It was shown for instance that upon aging IgA knockout mice tend to develop chronic airway inflammation that resembles chronic obstructive pulmonary disease (COPD) in humans (184). A COPD-like phenotype also develops in pIgR knockout mice upon exogenous bacterial challenge (185), and it has been shown in the past that COPD patients have an impaired pIgR expression (186) and reduced sIgA levels on the airway epithelium (187). Recently a similar phenomenon was reported for asthma (188) and rhinosinusitis (189). In the study of Ladjemi et al., asthmatics show a reduced immunostaining of pIgR in airway epithelia, with IL-4 and IL-13 being the suppressors of pIgR formation in the airway epithelium. Notably, there were no significant differences in the pIgR gene expression rate among asthmatics and healthy individuals (188). Thus, a posttranslational event such as proteolytic degradation of pIgR in the epithelium may be responsible for the observed differences.

A beneficial effect of allergen-specific IgA in the airway lumen was highlighted by Schwarze et al. (190). They showed that local application of a human monoclonal IgA antibody directed against the ragweed allergen Amb a attenuated the proinflammatory response to allergen inhalation in mice sensitized to Amb a I, whereas a control IgA against ovalbumin did not. Notably, the instilled anti-ragweed IgA induced the formation of Amb a I-specific IgG2a in the animals upon allergen challenge which indicates a shift toward Th1. Thus, IgA residing in the airways may have an anti-allergic/anti-asthmatic immunomodulatory activity. This effect may be explained by the IgA feedback loop, via which a secretory IgA response is adjusted to current needs. In order to provide an optimal defense against luminal noxa luminal IgA are continuously sampled at the epithelium and transported to the basolateral side, where it is inspected by immune cells whether it is loaded with antigen or not. If this is the case, an immune response is mounted or boosted (191). Although this type of transcytotic event has been attributed primarily to M cells, the set-up of the ragweed-study rather precludes that route in this specific case in as much as a human IgA1 against Amb a I was used and this type of IgA does not bind to murine M cells (172). However, with a plethora of IgA receptors known, other epithelial cell types may have taken over the task. The IgA-binding transferrin receptor, for instance, is expressed by type II pneumocytes and was shown to transport transferrin conjugates to the basolateral site (192). In addition, similar to the gut, airway dendritic cells, which also carry IgA receptors, send protrusions to the epithelial layer via which luminal antigen can be sampled (193, 194). Yet, sampling antigen-loaded IgA from the airway lumen and driving the airway immune response toward Th1 requires the presence of IgA in the lumen, which is reduced by the Th2 micro-milieu in allergic asthma. This results in a vicious circle of a locked-in Th2 environment where a lack of IgA causes a further lack of IgA.

This is in line with clinical observations on asthmatic patients that suggests a critical role for IgA in asthma pathogenesis. Patients with selective IgA deficiency tend to bronchial hyperresponsiveness (195) and children that show a delay in maturation of IgA production display atopic manifestations more often (196). Moreover, immunotherapy against the respective aeroallergen result in higher specific mucosal IgA levels along with lower skin prick test sensitivity (197) or lower airway hyperreactivity (198). Nevertheless, most of the above suggests a prominent role of sIgA or, more precisely, the lack thereof in the pathogenesis and chronicity of atopic asthma. Whether a lack of sIgA also plays a prominent role in asthma exacerbations remains to be elucidated.

## The Airway Epithelium as Mediator of an Acute Inflammatory Reaction

**FIGURE 3:**
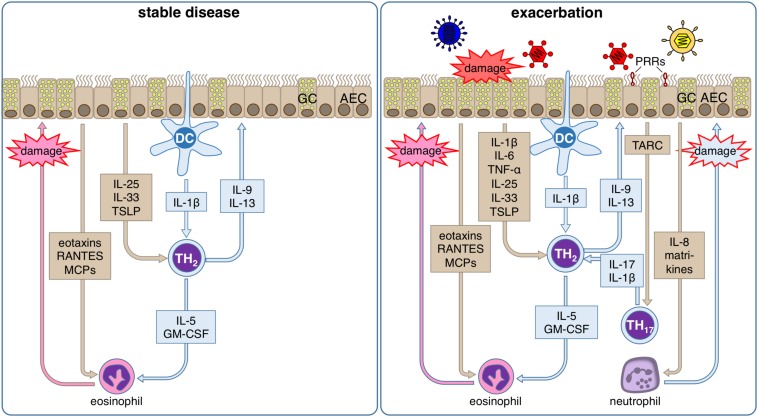
Inflammatory response of the airway epithelium during stable allergic asthma and exacerbation. During stable allergic asthma airway epithelial cells (AECs) release IL-25, IL-33 and TSLP supporting differentiation of T helper (TH) 2 cells that are activated by dendritic cells (DCs). Th2 cells in turn secrete IL-5 and GM-CSF that together with AEC-derived eotaxins, RANTES and MCPs regulate the production, maturation, recruitment and activation of eosinophils. Local degranulation of eosinophils in the lung eventually leads to damage of the airway epithelium. In parallel, the TH2-type cytokines IL-9 and IL-13 induce goblet cell (GC) metaplasia in airway epithelium. During viral induced asthma exacerbations, several other additional factors lead to an aggravation of this inflammatory response. Viral infection can be detected by the airway epithelium via pattern recognition receptors (PRR). Subsequently, AECs secrete on the one hand TARC, the main chemokine for the recruitment of Th17 cells that amplifies the proinflammatory effects of Th2 cells via release of IL-17, and on the other hand IL-8, which leads to the recruitment of neutrophils. Local degranulation of neutrophils in the lung eventually leads to additional damage of the airway epithelium. Viral infection of AECs also directly leads to damage of the airway epithelium. In summary, these conditions result in a markedly increased damage of the airway epithelium compared to the stable disease, which further impairs barrier integrity and leads to release of matrikines further amplifying the ongoing inflammation.

In addition to all the homeostatic defense functions like the maintenance of barrier integrity, transcytosis, and the mucosal clearance the airway epithelium also plays a major role against inhaled materials by producing several defense proteins such as mucins, defensins, antimicrobial peptides, cytokines, and chemokines (199). Thus, it contributes to local acute inflammatory reactions by regulating early inflammatory events via transcription and secretion of antimicrobial and pro-inflammatory proteins and by activating of mucin production (200, 201). Consequently, it is also a critical player during sensitization processes, asthma pathogenesis and acute exacerbations of the established disease (Figure 3).

## Danger and Pathogen Associated Molecular Pattern (Damp and Pamp) Recognition by Pattern Recognition Receptors (PRR) of the Airway Epithelium

Inhaled pathogens that are not cleared by MCC are recognized by airway epithelial cells (202). Equipped with a large number of PRRs such as cytoplasmic NOD like receptors (NLR) and transmembrane toll like receptors (TLR) that can respond to DAMPs and PAMPs AECs represent the first line of cells, which can respond to pathogens and other danger signals like cell stress and cell death in the lung (203).

DAMPs are molecules that are release from injured cells. Their presence is a clear sign for the loss of homeostatic integrity of specific cell compartments or even whole cells. Hence, they originate from the cytoplasm (S100 proteins, heat shock proteins, defensins, galectins, uric acid), the nucleus (High-Mobility-Group-Protein (HMGB1)), the endoplasmic reticulum (calreticulin), mitochondria (ATP, mitochondrial DNA, N-formylated peptides) or from the extracellular matrix (fibronectin, hyaluronan, versican) (204). Airway epithelial cells are both, responder to and producer of DAMPs (205, 206). The DNA binding protein HMGB1 for example, set free during necrosis of one cell can be detected from nearby cells by binding to their receptor AGE (RAGE), which leads to activation of nuclear factor kappa b (NFκB) and thereby to the production of pro-inflammatory mediators and consequently the recruitment of immune cells.

In turn, PAMPs are preserved molecules and structures from pathogens and toxins. They can originate from such different sources as bacteria, mycobacteria, viruses, fungi and parasites (207).

PAMPs and DAMPs activate signaling pathways resulting in the transcription and production of cytokines and chemokines. In brief, signal transduction through MyD88 and MyD88-independent mechanisms leads to the activation of NFκB, mitogen-activated protein (MAP) kinases, and interferon regulatory factor (IRF) 3 (203). Based on the nature of the triggering PAMPs and DAMPs and a possible preexisting inflammatory environment in the lung its signals can result in protective effects or pathological effects for the host organism (208). Repeated cell stress and exposure to pathogens trigger chronic activation of PRR pathways in airway epithelial cells that are highly active and play an important role in chronic airways diseases (204).

## PRR-Triggered Cytokine and Chemokine Reaction of the Airway Epithelium

Activation of PRRs by DAMPs leads to a massive secretion of proinflammatory mediators like IL-6, CXCL8, TNF that consequently entail infiltration of activated immune cells. Some of these cells like dendritic cells (DC), lymphocytes and mast cells are also involved in the pathogenesis of asthma (204, 209, 210). After contact for example with HDM extracts, representing a major source of asthma associated allergens, TLR4 dependent activation of NFκB and protease induced injuries in airway epithelial cells lead to secretion of chemokines and cytokines like thymic stromal lymphopoietin (TSLP), GM-CSF, IL-25, and IL-33 (211–215). This results in the activation and infiltration of DCs, innate lymphoid cells type 2 (ILC2) and Th2 cells (216–218).

During infection with bacterial pathogens airway epithelial cells can sense bacterial cell wall components via TLR2 (recognizing e.g., LTA), TLR4 (recognizing e.g., LPS), nucleotide-binding oligomerization domain-containing protein 1 (Nod1) and Nod2 (recognizing peptidoglycans) leading to activation of NFκB and subsequent immune responses and consequently to regulation of bacterial clearance (219, 220). Nucleic acid patterns arising during viral infection can be sensed via TLR3, TLR7/8, retinoic acid inducible gene I (RIG-1), and melanoma differentiation-associated protein 5 (MDA-5) (221–225). In response to TLR activation airway epithelial cells can also produce antimicrobial peptides such as human β-Defensin 2 (HBD-2) after TLR2 activation (201, 226).

The signals of different PRRs like TLRs, NLRs, and RAGE cooperate to regulate cellular immune responses to cell stress, infection and inflammation, which can amplify or dampen their effects (227).

Airway epithelial cells are very potent producers of cytokines and chemokines. The presence of aggressors like toxins and pathogens leads to production and fast and early secretion of IL-1β, IL-6, TNF, CXCL8, CCL11, and CCL20 (202, 228–230). Thereby, airway epithelial cells regulate and orchestrate local immunity by interacting with the recruitment of DCs, T-cells, and B-cells (CCL20), eosinophils (CCL11), and neutrophils (CXCL8). During viral infections they constitutively produce IFNβ to reduce viral replication and to support epithelial apoptosis (231). Thereby, airway epithelial cells represent the frontline of antiviral defense mechanisms. As mentioned earlier, increased concentrations of proinflammatory cytokines like IL-1β, IL-4, IL-13, and TNF can directly lead to damage of the barrier function of the airway epithelium (44–47, 232, 233).

In allergic asthma airway epithelial cells are one of the main producers of proinflammatory cytokines and chemokines like IL-13, IL-33, TSLP, CCL5 (Rantes), CCL7 (MCP-3), CCL17 (TARC), CCL22, and several eotaxins. All of these cytokines strongly direct or support the development of a Th2 polarized inflammation (234). The chemokines CCL17 and CCL22 play a prominent role in the recruitment of Th2 cells by binding to the CCR4 receptor, since activation of it is a key event for Th2 cell specific chemoattraction (235, 236). As a highly potent producer of TSLP the airway epithelium can create a local micro-milieu that supports and maintains a Th2 polarized inflammation (234, 237). In response to different epithelial injuries, airway epithelial cells secrete so-called alarmins like TSLP, IL-25, and IL-33 that direct T helper cell differentiation toward an Th2 phenotype (238). Additionally, secreted GM-CSF from airway epithelial cells leads to maturation and survival of eosinophils (239–241). Both effects are supporting allergic inflammation in asthma.

Taken together the airway epithelium plays a major role for the recognition of PAMPs and DAMPs in the lung. Binding of these molecules to their respective receptors enables the airway epithelium to regulate pathways important for barrier function, MCC and local immune responses. Functional disorders of the airway epithelium in the ability to answer the presence of PAMPs and DAMPs favor the development of chronic airways diseases. Viral infections and exposure to bacteria in early life modulate the acquisition of Th1 and Th2 immunity during further development and influence the responses to following exposures. These effects could play an even greater role in patients with asthma since they show disrupted MCC that could amplify the disease morbidity (242, 243). A cytokine induced Th2 polarization of the epithelium in combination with a barrier dysfunction induced by the same cytokines augments barrier impairment, further infiltration of proinflammatory cells, and enhanced penetration of inhaled allergens, which can be described as a self-reinforcing mechanism that predisposes for the development and perpetuation of allergic asthma (244–246).

Consequently, it has been suggested that an abnormal programming of the airway epithelium in general paired with an impaired capability to produce anti-inflammatory mediators such as IL-37 or α melanocyte stimulating hormone (α-MSH) may be the origin of chronic inflammatory airway diseases (247, 248).

## The Airway Epithelium in Viral Exacerbations of Asthma

Viral infections of the airways is of critical importance for the pathogenesis of allergic bronchial asthma: On the one hand recurrent respiratory viral infections during early childhood represent one of the strongest factors increasing the risk for the development of asthma in later life (249–253). On the other hand such infections are by far the most common cause for acute exacerbation of already established asthma leading to acute aggravation of disease symptoms and necessitating increased medication, GP visits, and can lead to hospitalization and critical care measures under certain conditions (254). Indeed, the airway epithelium is in the center of action during such an exacerbation, since it is not only the barrier that first comes into contact with viral pathogens, but its cells are also the target for their infection and the site of their replication. Thus, viral infection of the airway epithelium does not only impair the barrier integrity as already mentioned before, but also triggers the release of chemokines, cytokines, alarmins, and matrikines of the epithelial layer that affect the pre-existing inflammatory response in the asthmatic airway, which largely contributes to the formation of an acute exacerbation.

The viruses that have been implicated in asthma pathogenesis and especially the formation of acute exacerbation are HRV, RSV, influenza and parainfluenza viruses, human metapneumovirus, corona and adenoviruses, however, HRV infections appear to be the most common cause (255, 256). HRV is a non-enveloped, icosahedral virus, which belongs to the family of picornaviridae (genus enterovirus) and is subdivided into three clades (A, B, and C). The single-stranded positive RNA genome of HRV is constituted of ca. 7200 nucleotides (257, 258).

Clades A and B, which comprise the 100 most common serotypes, are further subdivided into a major and minor group. The major group utilizes the intracellular adhesion molecule-1 (ICAM-1) to bind to and transfect the host cell (259–261). The minor group HRV bind to the low density lipoprotein (LDL) receptor (262–265) and are considered to be more infectious. Clade C consists of 50 serotypes (266), which all bind to the cadherin-related family member 3 (CDHR3). All species of HRV have been shown to infect and replicate in airway epithelial cells (267).

Viral engagement with the specific receptor leads to transfection of the host cell (e.g., bronchial epithelial cells) and subsequently to a multitude of cellular responses. It is this cellular response that is believed to facilitate acute asthma exacerbation.

The cellular response to the virus is initiated by the detection of single-stranded RNA via TLRs -3, -7, and -8, MAD5, and RIG-I (221, 268). Activation of these receptors ultimately leads to the secretion of cytokines such as IL-1, IL-6, IL-8, IL-11, chemokines like CXCL10, CCL11 (eotaxin), CCL5 and anti-viral interferons of type I and III (268–274). The interferons have not only innate but also adaptive immune-system functionality to keep the viral infection locally at bay by mobilizing the adaptive immune response for effective viral clearance (275). Furthermore, proinflammatory cytokines like IL-1β and IL-6 are not specific for special types of immune response and thus, not only support the immune response against the invading virus but also promote the allergic immune response already established in the airways. Consequently, augmentation of the allergic immune response results in acute amplification of tissue damage, mucus production, and mediator release, and therefore in acute symptom aggravation.

Especially, the interferon response is thought to be an early post-infection event. In asthmatics epithelial interferon responses are believed to be hampered and as a consequence antiviral responses lack sufficient clearance (275). Not only interferons seem to be differently expressed in epithelial cells from asthmatics but also TSLP, which promotes Th2 responses (276). Another critical cytokine elevated in humans after HRV infections is IL-33, which also augments Th2 cell development (277).

## Airway Remodeling Is a Feature of Human Rhinovirus Infection

It is of note that viral infections not only initiate an immune response but also drive remodeling of the epithelial barrier and the subepithelial extracellular matrix (ECM). Hence, HRV16 induces perlecan, collagen V, tenascin c and matrix-associated (ma-) VEGF expression in an either TLR-3 or TLR-3/-7 associated manner *in vitro* (278, 279). Elevated expression of ma-VEGF and tenascin c was replicated in a mouse model of HRV infection, in which also Collagen I and fibronectin was found to be increased (278, 279). In addition, our group found human nasal epithelial cells infected with RV-16 *in vitro* to significantly downregulated genes associated with ECM receptor interaction and focal adhesion (280).

After infection and viral replication, the release of a vast array of mediators is among the earliest responses. In tissue culture a pneumocyte cell line expresses large amounts of IL-8 and CCL20 readily after 6 h post-infection (281). Also, the interferon response is thought to be an early post-infection event. In asthmatics epithelial interferon responses have been suggested to be hampered and as a consequence antiviral responses lack sufficient clearance (275). But not only interferons seem to be differently expressed in epithelial cells from asthmatics but also TSLP, which promotes Th2 responses (276). Another critical cytokine elevated in humans after HRV infections is IL-33. In line with that, Jackson et al. impressively showed, that the release of IL-33 by bronchial epithelial cells induces IL-4, IL-5, IL-13, and GATA3 expression in Th0 cells. An effect, which could be entirely blocked by an antibody against the IL-33 receptor (277).

Recently, active fragments from epithelial deposited ECM molecules have gained some recognition in asthma and asthma exacerbation. Matrikines are a class of molecules derived from ECM proteins (e.g., via proteolysis) with different properties from the parent molecule (282–284).

In 2010, Burgess et al. reported diminished levels of the collagen IV isoform alpha 3 (COL4A3) in airways from asthmatic subjects. The non-collagenous domain of COL4A3 is referred to as tumstatin and a biologically active matrikine. Treatment of mice with experimental allergic asthma with human recombinant tumstatin let to a significant reduction of hallmark disease features (e.g., airway hyperresponsiveness, ma-VEGF, eosinophil influx, IL13) (285). In another study, Van der Velden et al. identified an anti-angiogenic effect of tumstatin in a sheep model of asthma (286). Further investigations revealed a novel active region in Tumstatin (CP17), which significantly reduced neutrophil influx, mucus production in a mouse model of viral asthma exacerbation and reduced migrational speed and production of reactive oxygen species of neutrophils *in vitro* (287, 288). While the matrikine tumstatin conveys protection from experimental features of asthma and asthma exacerbation, a collagen I derived matrikine (PGP, Acetylated-(Ac) PGP) has been shown to be a more potent inducer of neutrophil chemotaxis then IL-8 and is found to be increased in severe asthmatics, a group of patients prone to develop exacerbations (283, 289).

Albeit ECM derived matrikines follow a different kinetical pathway (deposited first, released during inflammation) as compared to cytokines and chemokines (*de novo* production after viral infection), they can serve as protective or aggravating factors in asthma exacerbations.

In addition, the notion of epigenetic modification due to HRV infection in epithelial cells in asthma has gained attraction. McErlean et al. infected nasal epithelial cells from asthmatics and found evidence of reproducible changes to the methylome (290). We confirmed these results, which may unriddle how asthmatic airway epithelial cells may be able to respond differently to the same stimuli as compared to healthy epithelial cells (280). First studies to investigate this effect *in vivo* are underway (291, 292).

## E2-Polarized Airway Epithelium

Infection associated stimuli appear not to be the only factors that imprint mucosal immune reactions of the airway epithelium. In addition, the interaction between epithelial cells and leukocytes can lead to sustained alteration of respiratory epithelial cell biology. Even though these cells are definitely not able to constitute an immunological memory, it becomes more and more obvious that especially epithelial cells somehow memorize their exposure to certain environmental factors and the following insults and thereby develop some kind of trained immunity. We are just at the beginning to understand these processes and questions of which parts of the epithelium are trained and of how long the training effects sustain in the mucosa remain to be answered.

**FIGURE 4:**
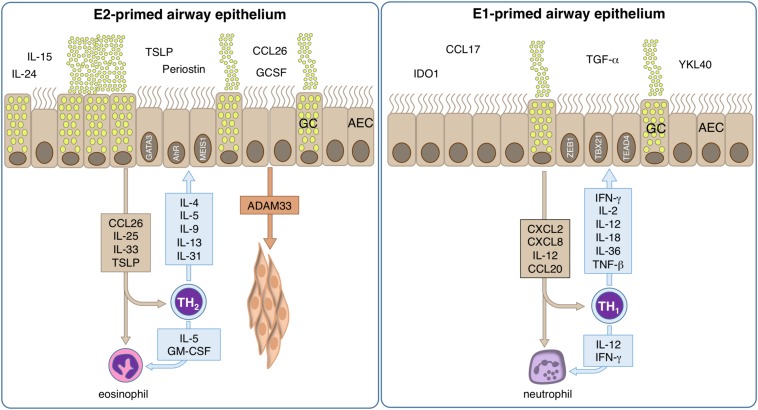
Mechanisms of allergic inflammation in epithelial cells. The role of tissue cells in the early phase of disease is largely unknown, but could provide important information about the pathologic development and could help to identify the causal relationships. However, bronchial epithelial cells are pre-committed to a type-2 (E2) or type-1 (E1) like phenotype. E2 epithelial cell activation by allergens takes place and their pro-inflammatory cytokines and chemokines induce inflammation and contribute to an epithelial type-2 response, so called “E2 response” with epithelial alarmins TSLP, IL-31, CCL-26, IL-25, and IL-33. Local type-2 responses involve multiple cytokines such as IL-4, IL-5, IL-9, IL-13, IL-25, IL-33, and increase of eosinophils. A series of chemokines are produced and migration of inflammatory cells to the allergic tissues takes place. The activation of e.g., smooth muscle cells by ADAM33 lead to remodeling. Bronchial hyperreactivity takes place leading to an enhanced susceptibility to bronchoconstriction. E1 epithelial cells respond to an infection releasing CXCL2, CXCL8, IL12 and CCL20, thus stimulating the local synthesis of IFN-γ, IL-2, IL-12, IL-18, IL-36, and TNF-α that present a wide range of antiviral activities, inducing up-regulation of MHC-I molecules and antiviral resistance in uninfected cells. Neutrophils respond to the infection signals IL-12 and IFN-γ by releasing pro-inflammatory cytokines, leading to the containment of the infection, rise of body temperature and to the recruitment of further phagocytic cells.

Thus, respiratory epithelial cells are constantly exposed to many types of challenges, including pathogens, allergens and environmental pollutants. Consequently, they are able to respond quickly and effectively to cellular damage such as the local cytokine production, lateral transport by ion exchanges, wide arrays of mucus compositions, secretion of antimicrobial peptides, and epithelial shedding. To date, it appears possible that different inflammatory environments as originated by for example typical Th1- or Th2-directed immune responses have a different impact on the biology of the respiratory epithelium and lead to some kind of E1- or E2- polarization of the respective epithelial cells (293) (Figure 4).

There is *in vivo* evidence for the inhibitory role of IFN-γ on asthma pathogenesis at the epithelial level indicating that type-1 responses counteract allergy (294, 295). The direct implication of airway epithelial cells was demonstrated by selective transgenic expression of the IFN-γ receptor on the airway epithelium and showed that IFN-γ inhibits mucus secretion, release of chitinases and eosinophilia independent of the activation of Th2 cells (296). In turn, GATA-3 inhibition causes an increase of T-BET und IFN-γ expression levels, leading to a dampened allergic phenotype (297). In addition, an increase in DNA-methylation of IFN-γ was observed during allergic sensitization (298), while perinatal prevention of allergy mediated by *Acinetobacter* does not show the anticipated drop in H4 acetylation in the IFN-γ promoter (299). The immunological consequence of epithelial differentiation becomes increasingly interesting, as sensitization, but also recovery processes and airway remodeling could open new options for intervention and prevention of lung damage. Increasing evidence of mechanisms involving epithelial cytokine production such as CCL-26, and the epithelium-derived alarmins TSLP and IL-33 are substantiating the current focus on the cross talk between airway epithelium and immune cells in allergy research.

However, it is unknown whether epithelial cells are influenced by IL-4 prior or with entry into terminal differentiation. This early influence could imprint the offspring cells that populate the epithelial surface and therefore have major consequences for the physiology of the airways. IL-4 was shown to have a major effect on the epithelium, as mice overexpressing this cytokine under the Club cell-secretory protein 10 (CC10) promoter show increased cellular infiltration, epithelial hypertrophy, mucus cell hyperplasia, secretion of gastric mucins and surfactant proteins (SP) A and B (300). While this model effectively demonstrated all hallmarks of experimental allergic asthma, it did not demonstrate whether IL-4 itself is inducing differentiation of basal cells or whether secondary effects trigger epithelial differentiation. However, the differentiation effects could also be observed in human primary epithelial cells of the nose, where IL-13 modulates the differentiation toward less ciliated and more secretory cells (114). To date, it is controversially discussed, whether IL-4 and IL-13 can also affect fully differentiated epithelial cells in air liquid-interphase cultures or whether this is only possible in immature submerged cultures (301, 302). However, during the epithelial differentiation process induced by air-liquid exposure, the addition of IL-4 enhances expression of certain antimicrobial peptides (303) and eicosanoids (304). Furthermore, it was demonstrated that IL-4 and IL-13, through inhibition of TLR3 expression and signaling (IRF3), impair immune responses to HRV infection (305). This is in line with the finding that chronic house dust mite exposure in the airways not only causes a strong Th2-directed inflammation but also diminishes anti-rhinovirus responses and local IFN expression, particularly of epithelial IFN-λ (306). In line with this, transgenic IL-4 expression in the lungs reduces cytotoxic T cell responses against Influenza viruses (307). On the level of secreted factors, it was shown that cytokines such as Wnt5a (Wingless-Type MMTV Integration Site Family, Member 5a) or IL-24 are expressed as response to IL-4 stimulation only, while proteins with known pathological roles such as the IL-4 induced protein CCL-26 or periostin were shown to be upregulated by IL-4 and down-regulated in IFN-γ environment. These results were consistent when comparing upper and lower airway secretions, thus confirming nasal lining fluids as a proxy for the lower respiratory tract, particularly for epithelial type-2 biomarker like CCL-26 and IL24 (308, 309). The E2-related transcription factor network contained the E2 hub-transcription factors *GATA3, NFE2, MEIS1, HEY2*, and *AHR*: *GATA3* is well known as the master transcription factor of type-2 response in immune cells, however it was also shown to be expressed in airway epithelial cells. *NFE2* was demonstrated to have a cytoprotective activity against epithelial cell injury by cigarette smoke, which could hint on a protective role in an IL-4 dominated micro-milieu (310). For *MEIS1*, it has been demonstrated that its inactivation produces an increase in airway smooth muscle mass and a corresponding decrease in cartilage and suggesting an important role in allergic airway diseases. A loss of Hox gene function, however, does not preclude airway repair, but regenerated epithelium displays goblet cell metaplasia and less SCGB1A1-positive cells, demonstrating the essential role of Hoxa5 for correct differentiation. This goblet cell metaplasia is further associated with increased Notch signaling activity. Consistent with these findings, expression levels of activated NOTCH1 and the effector gene HEY2 are in turn enhanced in patients with allergic disease (293, 311, 312). Taken together, E2-polarization has a marked impact on the barrier and especially immune functions of the airway epithelium and at least supports impairment of the MCC and antiviral responses, both factors that are critical in asthma pathogenesis.

## Conclusion

In summary, the airway epithelium exerts a broad variety of immune functions that range from passive barrier over MCC, active production and transport of pathogen-neutralizing molecules to pathogen recognition and targeting as well as cytokine and chemokine release. The airway epithelium is usually the first tissue that is exposed to inhaled allergens, pathogens or pollutants. Since it is able to react on this contact by inducing local inflammatory reactions, it is clearly a central part of the local immune response and bridges innate and adaptive immune functions against all types of harmful intruders entering the respiratory system. Therefore, the airway epithelium is a key factor in asthma pathogenesis and plays a critical role in the development as well as in the progression and exacerbation of the disease: Hence, a disturbed cellular barrier enables allergens to enter the body and to induce a sensitization reaction, which is widely regarded as the starting point of an asthma career. Down the line, the protective mucus and PCL layers provided by the epithelium as physical barriers are compromised and the release of pathogen deterring molecules such as secretory Iimmunoglobulins becomes impaired. As a consequence, this frontline toward invading pathogens can be breached and airway epithelial cells are infected and even destroyed by respiratory pathogens. A vicious cycle is started where barrier disturbance and infection promote each other. The latter, in particular with certain viruses, is also one of the strongest factors predisposing toward asthma development in early childhood.

Once asthma has been established, the airway epithelium responds to further viral infection with the release of manifold factors promoting not only the antiviral response but also augmenting the already present allergic inflammation and thus promoting acute asthma exacerbations. Finally yet importantly, it is also impaired and polarized by products released from cells of the chronified allergic immune response in the airways, which leads to impairment of mucus production, MCC, and the anti-inflammatory IFN-response in E2-polarized airway epithelial cells. Recent studies indicate that, in addition to this E2-polarization, also the history of allergy- and pathogen-derived insults does not only have a transient effect on the airway epithelium, but leaves some kind of memory in these cells that can be described as “imprinting” or “trained immunity.” Understanding the mechanisms underlying these processes would not only help to further understand the key role of airway epithelial cells in asthma pathogenesis but also could identify new targets for the treatment or even prevention of allergic asthma.

## Author Contributions

All authors listed have made a substantial, direct and intellectual contribution to the work, and approved it for publication.

## Conflict of Interest

The authors declare that the research was conducted in the absence of any commercial or financial relationships that could be construed as a potential conflict of interest.
